# (1*R*,3*R*,4*R*,6*S*)-4-(7-Meth­oxy-2-oxo-2*H*-chromen-6-yl)-1-methyl-3,6-dioxa­bicyclo­[3.1.0]hexan-2-yl acetate

**DOI:** 10.1107/S1600536812047617

**Published:** 2012-11-24

**Authors:** Wong Phakhodee, Surat Laphookhieo, Timothy John Prior, Apinpus Rujiwatra

**Affiliations:** a Natural Products Research Laboratory, School of Science, Mae Fah Luang, University, Tasud, Muang, Chiang Rai 57100, Thailand; b Department of Chemistry, University of Hull, Cottingham Road, Hull HU6 7RX, England; c Department of Chemistry, Faculty of Science, Chiang Mai University, Chiang Mai 50200, Thailand

## Abstract

In the title compound, C_17_H_16_O_7_, which was isolated from the leaves of *Micromelum integerrimum*, the furan ring adopts an envelope conformation with the O atom as the flap. An intra­molecular C—H⋯O hydrogen bond occurs. The carbonyl O atom is disordered in a 0.57 (8):0.43 (8) ratio. In the crystal, mol­ecules are linked by weak C—H⋯O hydrogen bonds into a *C*(10) chain along [010].

## Related literature

*Micromelum integerrimum* is a shrub in the Rutacae family containing the coumarin mol­ecule, micromelin, as the major chemical constituent (Cassady *et al.*, 1979 ▶). Many coumarins including micromelin have been extracted from Rutacae plants, and for some their cytotoxicity has been investigated (Sripisut *et al.*, 2012 ▶; He *et al.*, 2001 ▶). For previous reports on the isolation of micromelin (micromelumin) from a Northern Queensland collection, an Assamese collection, and a Northeast Thailand collection, see: Lamberton *et al.* (1967 ▶); Das *et al.* (1984 ▶); Siridechakorn *et al.* (2012 ▶). For detailed H^1^ NMR spectroscopic data, see: Das *et al.* (1984 ▶); Siridechakorn *et al.* (2012 ▶). For a phytochemical investigation, see: Siridechakorn *et al.* (2012 ▶). For a closely related micromelin structure, C_15_H_12_O_6_, see: Fun *et al.* (2011 ▶). For hydrogen-bond motifs, see: Bernstein *et al.* (1995 ▶). For puckering parameters, see: Cremer & Pople (1975 ▶).
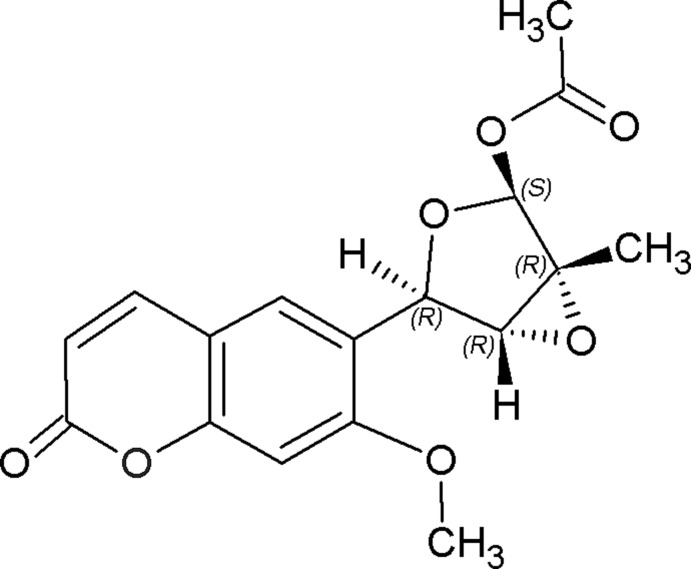


## Experimental

### 

#### Crystal data

C_17_H_16_O_7_
*M* *_r_* = 332.31
Monoclinic, 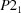

*a* = 10.4825 (16) Å
*b* = 6.9213 (9) Å
*c* = 11.0212 (18) Å
β = 95.970 (7)°
*V* = 795.3 (2) Å^3^
*Z* = 2
Mo *K*α radiation
μ = 0.11 mm^−1^
*T* = 298 K
0.64 × 0.32 × 0.24 mm

#### Data collection

Bruker SMART APEXII CCD area-detector diffractometer
Absorption correction: multi-scan (*SADABS*; Bruker, 1997 ▶) *T*_min_ = 0.653, *T*_max_ = 0.746
4381 measured reflections
2123 independent reflections
1692 reflections with *I* > 2σ(*I*)
*R*_int_ = 0.021

#### Refinement

*R*[*F* ^2^ > 2σ(*F* ^2^)] = 0.046
*wR*(*F* ^2^) = 0.136
*S* = 1.06
2123 reflections
223 parameters
2 restraints
H atoms treated by a mixture of independent and constrained refinement
Δρ_max_ = 0.47 e Å^−3^
Δρ_min_ = −0.24 e Å^−3^

### 

Data collection: *APEX2* (Bruker, 2008 ▶); cell refinement: *SAINT* (Bruker, 1997 ▶); data reduction: *SAINT*; program(s) used to solve structure: *SHELXS97* (Sheldrick, 2008 ▶); program(s) used to refine structure: *SHELXL97* (Sheldrick, 2008 ▶); molecular graphics: *DIAMOND* (Brandenburg, 2007 ▶); software used to prepare material for publication: *publCIF* (Westrip, 2010 ▶) and *PLATON* (Spek, 2009 ▶).

## Supplementary Material

Crystal structure: contains datablock(s) I, global. DOI: 10.1107/S1600536812047617/bx2427sup1.cif

Structure factors: contains datablock(s) I. DOI: 10.1107/S1600536812047617/bx2427Isup2.hkl

Additional supplementary materials:  crystallographic information; 3D view; checkCIF report

## Figures and Tables

**Table 1:** Hydrogen-bond geometry (Å, °)

*D*—H⋯*A*	*D*—H	H⋯*A*	*D*⋯*A*	*D*—H⋯*A*
C4—H4⋯O19*A* ^i^	1.07 (3)	2.46 (2)	3.064 (8)	114 (1)
C5—H5⋯O13	1.01 (3)	2.58 (3)	3.403 (3)	139 (1)
C12—H12⋯O2^ii^	0.98	2.35	3.282 (5)	158
C16—H16*B*⋯O2^iii^	0.96	2.54	3.419 (4)	153

Symmetry codes: (i) 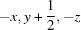; (ii) 
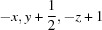
; (iii) 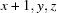.

**Fig. 1.**
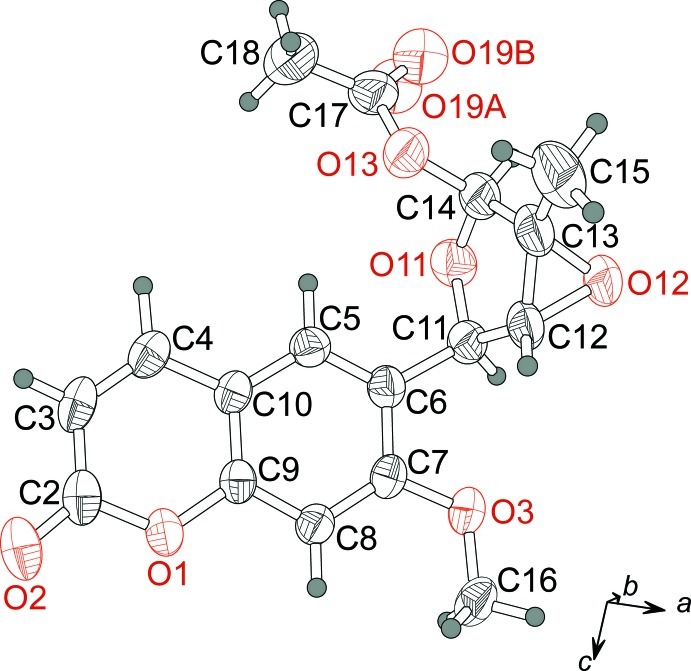
Molecular structure of the title compound. Displacement ellipsoids are drawn at 50% probability level.

**Fig. 2.**
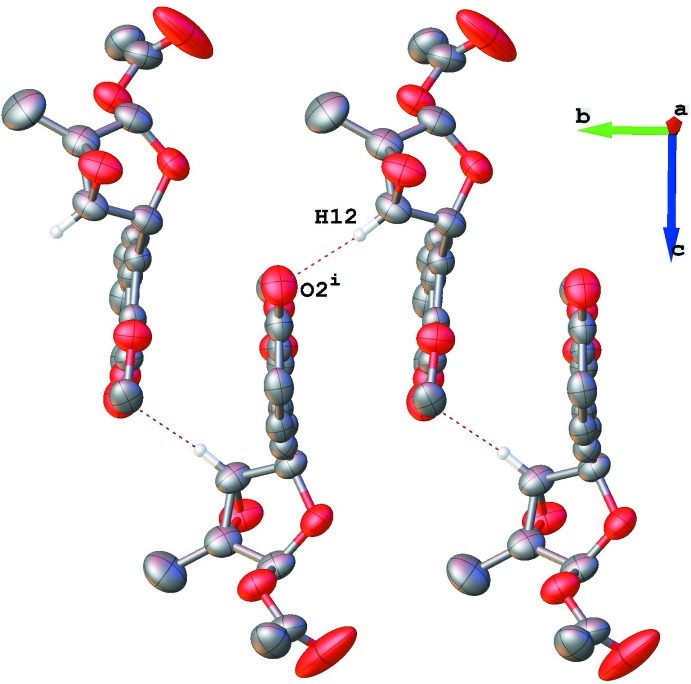
A view of (I), showing the C(10) chains along [010] constructed via C—H···O hydrogen bonds. Hydrogen bonds are depicted as dashed lines [symmetry-code:(i) -x,1/2+y, 1-z]

## supplementary crystallographic information

### Comment

*Micromelum integerrimum* is a shrub in the Rutacae family containing the coumarin molecule, micromilin, as the major chemical constituent (Cassady *et al.*, 1979). Many coumarins including micromilin have been extracted from Rutacae plants, and for some their cytotoxicity has been investigated (Sripisut *et al.*, 2012; He *et al.*, 2001). In the attempt to investigate the chemical constitutents of the extract of *Micromelum integerrimum* leaves collected from Chiang Rai province in the Northern part of Thailand (March 2012), the colourless crystals of the title compound have been isolated and examined.

The absolute configurations of the four chiral centres in the molecule, (I) (C11, C12, C13, and C14) were assigned from a previous report (Das *et al.*, 1984) as *R*, *R*, *R* and *S*, respectively. The benzene and dihydropyran ring system (C2–C10/O1) and also the carboxyl O2 and the methoxy O3 atoms are co-planar with the *r.m.s.* 0.004 (3) Å (Spek, 2009). A deviation of atoms O2 and O3 from the benzene and dihydropyran ring system are 0.028 (2) Å and 0.040 (2) Å, respectively. The five-membered furan ring (C11–C14/O11) shows envelope conformation with the puckering atom O11 of 0.075 (2) Å, and forms an angle of 66.11 (9)° to the twelve-membered benzene–dihydropyran due to free rotation about the C6—C11 bond. The puckering parameters Q and φ of the furan ring are 0.116 (2) Å and 176.4 (12)°, respectively (Cremer & Pople, 1975). The orientation of the oxiran ring (C12–C13/O12) attached to the furan can be defined by the dihedral angle being 79.5 (2)° with atom O12 of the oxirane ring located 1.250 (2) Å away from the furan plane. Regarding the acetate group, atom O19 shows minor positional disorder over two sites. The acetate O13 is arranged at 64.19 (15)° in reference to the furan plane, and the torsion angle measured on C14–O13–C17–C18 is 171.6 (2)°.In the crystal of (I), the molecules are linked by weak C—H···O hydrogen-bonding interactions into a chain along [010] with set-graph notation C(10), (Bernstein, *et al.*, 1995)

### Experimental

The title compound was obtained from an acetone extract of *Micromelum integerrimum* leaves (0.55 kg), which are collected from Chiang Rai Province, Thailand. From seven fractions (A—G) yielded by column chromatography using hexanes-acetone, the title compound (295.9 mg) was isolated from fraction D by also column chromatography using 2% acetone-CH_2_Cl_2_. The crystals were then crystallized by slowly evaporation of the solvent.

### Refinement

The carbonyl group O atom is disordered over two sets of sites in a 0.57 (8):0.43 (8) ratio.Friedel opposites were merged in the final cycles of refinement as there is no appreciable anomalous scattering at the wavelength used for data collection. H atoms were placed in geometrically idealized positions (C—H= 0.91-1.06 Å, C(methyl)—H=0.96Å and were constrained to ride on their parects atoms with U_iso_(H)= 1.2U_eq_(C) and 1.5U_eq_(C) respectively , except H3, H4, H5, and H8, were refined freely, with isotropic displacement parameters.

### Crystal data

**Table:**

C_17_H_16_O_7_	*F*(000) = 348
*M**_r_* = 332.31	*D*_x_ = 1.388 Mg m^−^^3^
Monoclinic, *P*2_1_	Mo *K*α radiation, λ = 0.71073 Å
Hall symbol: P 2yb	Cell parameters from 3003 reflections
*a* = 10.4825 (16) Å	θ = 1.9–28.3°
*b* = 6.9213 (9) Å	µ = 0.11 mm^−^^1^
*c* = 11.0212 (18) Å	*T* = 298 K
β = 95.970 (7)°	Block, colourless
*V* = 795.3 (2) Å^3^	0.64 × 0.32 × 0.24 mm
*Z* = 2	

### Data collection

**Table:**

Bruker SMART APEXII CCD area-detector diffractometer	2123 independent reflections
Radiation source: fine-focus sealed tube	1692 reflections with *I* > 2σ(*I*)
Graphite monochromator	*R*_int_ = 0.021
ω scans	θ_max_ = 28.3°, θ_min_ = 2.6°
Absorption correction: multi-scan (*SADABS*; Bruker, 1997)	*h* = −13→8
*T*_min_ = 0.653, *T*_max_ = 0.746	*k* = −7→9
4381 measured reflections	*l* = −14→14

### Refinement

**Table:**

Refinement on *F*^2^	Primary atom site location: structure-invariant direct methods
Least-squares matrix: full	Secondary atom site location: difference Fourier map
*R*[*F*^2^ > 2σ(*F*^2^)] = 0.046	Hydrogen site location: inferred from neighbouring sites
*wR*(*F*^2^) = 0.136	H atoms treated by a mixture of independent and constrained refinement
*S* = 1.06	*w* = 1/[σ^2^(*F*_o_^2^) + (0.0701*P*)^2^ + 0.1456*P*] where *P* = (*F*_o_^2^ + 2*F*_c_^2^)/3
2123 reflections	(Δ/σ)_max_ < 0.001
223 parameters	Δρ_max_ = 0.47 e Å^−^^3^
2 restraints	Δρ_min_ = −0.24 e Å^−^^3^

### Special details

**Table:**

Geometry. All e.s.d.'s (except the e.s.d. in the dihedral angle between two l.s. planes) are estimated using the full covariance matrix. The cell e.s.d.'s are taken into account individually in the estimation of e.s.d.'s in distances, angles and torsion angles; correlations between e.s.d.'s in cell parameters are only used when they are defined by crystal symmetry. An approximate (isotropic) treatment of cell e.s.d.'s is used for estimating e.s.d.'s involving l.s. planes.
Refinement. Refinement of *F*^2^ against ALL reflections. The weighted *R*-factor *wR* and goodness of fit *S* are based on *F*^2^, conventional *R*-factors *R* are based on *F*, with *F* set to zero for negative *F*^2^. The threshold expression of *F*^2^ > σ(*F*^2^) is used only for calculating *R*-factors(gt) *etc*. and is not relevant to the choice of reflections for refinement. *R*-factors based on *F*^2^ are statistically about twice as large as those based on *F*, and *R*-factors based on ALL data will be even larger.

### Fractional atomic coordinates and isotropic or equivalent isotropic displacement parameters (Å^2^)

**Table:**

	*x*	*y*	*z*	*U*_iso_*/*U*_eq_	Occ. (<1)
O1	−0.09274 (16)	0.0334 (4)	0.56547 (17)	0.0485 (5)	
C2	−0.2211 (2)	0.0363 (5)	0.5224 (3)	0.0509 (6)	
O2	−0.2958 (2)	0.0392 (4)	0.5982 (2)	0.0701 (7)	
C3	−0.2509 (2)	0.0355 (5)	0.3921 (3)	0.0542 (7)	
H3	−0.335 (3)	0.0402 (6)	0.3602 (13)	0.065*	
C4	−0.1595 (2)	0.0280 (5)	0.3155 (3)	0.0490 (6)	
H4	−0.1858 (8)	0.0256 (5)	0.219 (3)	0.059*	
C5	0.0765 (2)	0.0093 (4)	0.2907 (2)	0.0434 (6)	
H5	0.0579 (6)	0.0024 (5)	0.199 (3)	0.052*	
C6	0.2015 (2)	0.0050 (4)	0.3416 (2)	0.0438 (6)	
C7	0.2260 (2)	0.0189 (4)	0.4699 (2)	0.0425 (5)	
O3	0.35235 (16)	0.0218 (4)	0.51324 (18)	0.0587 (6)	
C16	0.3858 (3)	0.0418 (6)	0.6410 (3)	0.0572 (7)	
H16A	0.3431	0.1526	0.6702	0.086*	
H16B	0.4769	0.0582	0.6572	0.086*	
H16C	0.3600	−0.0719	0.6820	0.086*	
C8	0.1272 (2)	0.0280 (5)	0.5436 (2)	0.0423 (5)	
H8	0.1452 (6)	0.0344 (5)	0.633 (3)	0.051*	
C9	0.0026 (2)	0.0280 (4)	0.4886 (2)	0.0389 (5)	
C10	−0.0266 (2)	0.0232 (4)	0.3624 (2)	0.0408 (5)	
C11	0.3156 (3)	−0.0214 (5)	0.2692 (3)	0.0502 (7)	
H11	0.3788	−0.1042	0.3159	0.060*	
O11	0.2824 (2)	−0.1069 (4)	0.1510 (2)	0.0606 (6)	
C12	0.3783 (3)	0.1663 (6)	0.2434 (3)	0.0563 (8)	
H12	0.3774	0.2751	0.3003	0.068*	
O12	0.48617 (19)	0.1365 (5)	0.1725 (2)	0.0697 (7)	
C13	0.3647 (3)	0.1984 (6)	0.1116 (3)	0.0621 (8)	
C14	0.2937 (3)	0.0253 (6)	0.0571 (3)	0.0599 (8)	
H14	0.3396	−0.0323	−0.0070	0.072*	
O13	0.1675 (2)	0.0904 (4)	0.00763 (19)	0.0656 (7)	
C15	0.3633 (4)	0.3895 (9)	0.0470 (4)	0.0931 (14)	
H15A	0.2762	0.4289	0.0248	0.140*	
H15B	0.4066	0.3773	−0.0252	0.140*	
H15C	0.4062	0.4845	0.1000	0.140*	
C17	0.1085 (3)	−0.0092 (6)	−0.0852 (3)	0.0652 (9)	
C18	−0.0245 (4)	0.0466 (8)	−0.1189 (4)	0.0793 (11)	
H18A	−0.0547	−0.0112	−0.1957	0.119*	
H18B	−0.0301	0.1847	−0.1258	0.119*	
H18C	−0.0762	0.0031	−0.0573	0.119*	
O19A	0.1483 (6)	−0.1679 (10)	−0.1149 (6)	0.0843 (12)*	0.567 (8)
O19B	0.1763 (7)	−0.0896 (14)	−0.1535 (7)	0.0843 (12)*	0.433 (8)

### Atomic displacement parameters (Å^2^)

**Table:**

	*U*^11^	*U*^22^	*U*^33^	*U*^12^	*U*^13^	*U*^23^
O1	0.0370 (8)	0.0543 (11)	0.0556 (10)	0.0019 (10)	0.0112 (7)	0.0022 (11)
C2	0.0374 (12)	0.0414 (14)	0.0750 (18)	0.0000 (13)	0.0113 (12)	0.0053 (16)
O2	0.0463 (10)	0.0742 (16)	0.0943 (16)	−0.0031 (13)	0.0283 (10)	0.0057 (15)
C3	0.0318 (11)	0.0494 (15)	0.0794 (19)	−0.0042 (14)	−0.0037 (11)	0.0071 (17)
C4	0.0404 (12)	0.0459 (14)	0.0585 (15)	−0.0051 (14)	−0.0054 (11)	0.0033 (15)
C5	0.0440 (12)	0.0436 (14)	0.0424 (12)	−0.0009 (12)	0.0030 (10)	0.0011 (12)
C6	0.0382 (11)	0.0446 (15)	0.0489 (13)	0.0013 (12)	0.0067 (10)	−0.0025 (13)
C7	0.0328 (10)	0.0427 (13)	0.0513 (13)	0.0040 (12)	0.0007 (9)	0.0008 (13)
O3	0.0323 (8)	0.0833 (16)	0.0599 (11)	0.0021 (12)	0.0015 (7)	−0.0034 (13)
C16	0.0388 (12)	0.0655 (19)	0.0640 (16)	0.0071 (15)	−0.0103 (11)	−0.0031 (17)
C8	0.0380 (11)	0.0451 (13)	0.0433 (12)	0.0010 (13)	0.0016 (9)	−0.0007 (13)
C9	0.0344 (10)	0.0341 (11)	0.0486 (12)	0.0009 (12)	0.0059 (9)	0.0029 (12)
C10	0.0354 (10)	0.0373 (12)	0.0491 (12)	−0.0022 (12)	0.0006 (9)	0.0003 (12)
C11	0.0416 (13)	0.0589 (18)	0.0509 (14)	0.0051 (13)	0.0080 (11)	−0.0103 (13)
O11	0.0620 (13)	0.0600 (12)	0.0615 (13)	0.0007 (11)	0.0147 (10)	−0.0160 (11)
C12	0.0384 (13)	0.075 (2)	0.0578 (16)	−0.0085 (15)	0.0171 (12)	−0.0156 (16)
O12	0.0379 (10)	0.110 (2)	0.0636 (13)	−0.0054 (12)	0.0170 (9)	−0.0161 (14)
C13	0.0450 (15)	0.082 (2)	0.0624 (18)	−0.0085 (17)	0.0210 (13)	−0.0089 (18)
C14	0.0442 (13)	0.083 (2)	0.0541 (15)	0.0060 (18)	0.0143 (11)	−0.0172 (18)
O13	0.0501 (11)	0.0877 (18)	0.0588 (12)	0.0055 (11)	0.0046 (9)	−0.0196 (12)
C15	0.081 (3)	0.104 (3)	0.099 (3)	−0.023 (3)	0.030 (2)	0.021 (3)
C17	0.0663 (18)	0.083 (3)	0.0468 (14)	0.0084 (18)	0.0062 (13)	−0.0103 (16)
C18	0.071 (2)	0.092 (3)	0.072 (2)	−0.001 (2)	−0.0103 (17)	0.006 (2)

### Geometric parameters (Å, º)

**Table:**

O1—C9	1.377 (3)	C11—O11	1.441 (3)
O1—C2	1.380 (3)	C11—C12	1.496 (5)
C2—O2	1.203 (3)	C11—H11	0.9800
C2—C3	1.438 (4)	O11—C14	1.396 (5)
C3—C4	1.343 (4)	C12—O12	1.455 (3)
C3—H3	0.9168	C12—C13	1.463 (4)
C4—C10	1.435 (3)	C12—H12	0.9800
C4—H4	1.0676	O12—C13	1.441 (4)
C5—C6	1.371 (3)	C13—C15	1.501 (7)
C5—C10	1.406 (3)	C13—C14	1.502 (5)
C5—H5	1.0053	C14—O13	1.450 (4)
C6—C7	1.413 (4)	C14—H14	0.9800
C6—C11	1.517 (3)	O13—C17	1.332 (4)
C7—O3	1.361 (3)	C15—H15A	0.9600
C7—C8	1.383 (3)	C15—H15B	0.9600
O3—C16	1.422 (3)	C15—H15C	0.9600
C16—H16A	0.9600	C17—O19B	1.222 (7)
C16—H16B	0.9600	C17—O19A	1.231 (7)
C16—H16C	0.9600	C17—C18	1.456 (5)
C8—C9	1.382 (3)	C18—H18A	0.9600
C8—H8	0.9857	C18—H18B	0.9600
C9—C10	1.393 (4)	C18—H18C	0.9600
			
C9—O1—C2	122.2 (2)	C6—C11—H11	108.7
O2—C2—O1	116.3 (3)	C14—O11—C11	111.7 (3)
O2—C2—C3	127.2 (3)	O12—C12—C13	59.20 (19)
O1—C2—C3	116.5 (2)	O12—C12—C11	111.1 (3)
C4—C3—C2	122.2 (2)	C13—C12—C11	108.8 (3)
C4—C3—H3	118.9	O12—C12—H12	120.8
C2—C3—H3	118.9	C13—C12—H12	120.8
C3—C4—C10	120.3 (2)	C11—C12—H12	120.8
C3—C4—H4	119.9	C13—O12—C12	60.65 (19)
C10—C4—H4	119.9	O12—C13—C12	60.14 (19)
C6—C5—C10	121.9 (2)	O12—C13—C15	116.6 (3)
C6—C5—H5	119.0	C12—C13—C15	126.9 (4)
C10—C5—H5	119.0	O12—C13—C14	109.0 (3)
C5—C6—C7	118.4 (2)	C12—C13—C14	105.6 (3)
C5—C6—C11	124.1 (2)	C15—C13—C14	122.2 (3)
C7—C6—C11	117.6 (2)	O11—C14—O13	109.8 (2)
O3—C7—C8	123.6 (2)	O11—C14—C13	107.6 (3)
O3—C7—C6	115.0 (2)	O13—C14—C13	107.3 (3)
C8—C7—C6	121.4 (2)	O11—C14—H14	110.7
C7—O3—C16	118.7 (2)	O13—C14—H14	110.7
O3—C16—H16A	109.5	C13—C14—H14	110.7
O3—C16—H16B	109.5	C17—O13—C14	117.4 (3)
H16A—C16—H16B	109.5	C13—C15—H15A	109.5
O3—C16—H16C	109.5	C13—C15—H15B	109.5
H16A—C16—H16C	109.5	H15A—C15—H15B	109.5
H16B—C16—H16C	109.5	C13—C15—H15C	109.5
C9—C8—C7	118.3 (2)	H15A—C15—H15C	109.5
C9—C8—H8	120.9	H15B—C15—H15C	109.5
C7—C8—H8	120.9	O19B—C17—O13	117.1 (4)
O1—C9—C8	116.3 (2)	O19A—C17—O13	121.5 (4)
O1—C9—C10	121.2 (2)	O19B—C17—C18	124.5 (4)
C8—C9—C10	122.5 (2)	O19A—C17—C18	120.7 (4)
C9—C10—C5	117.4 (2)	O13—C17—C18	114.4 (3)
C9—C10—C4	117.6 (2)	C17—C18—H18A	109.5
C5—C10—C4	125.0 (2)	C17—C18—H18B	109.5
O11—C11—C12	104.7 (2)	H18A—C18—H18B	109.5
O11—C11—C6	113.3 (2)	C17—C18—H18C	109.5
C12—C11—C6	112.4 (2)	H18A—C18—H18C	109.5
O11—C11—H11	108.7	H18B—C18—H18C	109.5
C12—C11—H11	108.7		

### Hydrogen-bond geometry (Å, º)

**Table:**

*D*—H···*A*	*D*—H	H···*A*	*D*···*A*	*D*—H···*A*
C4—H4···O19*A*^i^	1.07 (3)	2.46 (2)	3.064 (8)	114 (1)
C5—H5···O13	1.01 (3)	2.58 (3)	3.403 (3)	139 (1)
C12—H12···O2^ii^	0.98	2.35	3.282 (5)	158
C16—H16*B*···O2^iii^	0.96	2.54	3.419 (4)	153

Symmetry codes: (i) −*x*, *y*+1/2, −*z*; (ii) −*x*, *y*+1/2, −*z*+1; (iii) *x*+1, *y*, *z*.
